# Touch-Typing Detection Using Eyewear: Toward Realizing a New Interaction for Typing Applications †

**DOI:** 10.3390/s19092022

**Published:** 2019-04-30

**Authors:** Tatsuhito Hasegawa, Tatsuya Hatakenaka

**Affiliations:** Department of Information Science, Faculty of Engineering, University of Fukui, Fukui 910-8507, Japan; material.80s@gmail.com

**Keywords:** touch-typing, JINS MEME, touch-typing skills estimation

## Abstract

Typing skills are important in the digital information society of this generation. As a method to improve typing speed, in this study, we focused on the training of touch typing that enables typing a key without looking at the keyboard. For support of touch-typing training, it is efficient to apply a penalty if a learner looks at the keyboard; however, to realize the penalty method, the computer needs to be able to recognize whether the learner looked at the keyboard. We, therefore, proposed a method to detect a learner’s eye gaze, namely, using eyewear to detect whether the learner looked at the keyboard, and then evaluating the detection accuracy of our proposed method. We examined the necessity for our system by analyzing the relationship between a learner’s eye gaze and touch-typing skills.

## 1. Introduction

Due to the ubiquitous nature of information technology, typing skills have become more important than ever. With the spread of smartphones and tablets, people can easily access the Internet now, anytime, anywhere. Although many people own smartphones, they also use conventional PCs in their daily lives, for one reason, because of the ease of character input. In general, the character input speed using a PC keyboard is faster than the character input speed on a smartphone. Additionally, considering the use of computers in business, typing skills have become necessary for a wide range of activities, such as e-mailing, filling out paperwork, preparing documents and presentation materials, and writing dissertations, to name just a few. By improving typing speeds, work performance may be improved. In addition, better typing speeds can have an impact on operational efficiency.

To support typing skills improvement, companies and individuals developed many typing training applications. For example, TypingClub (https://www.typingclub.com/)) prepares typing lessons according to the user’s stage of skill development. To enhance learners’ engagement, many systems incorporate gaming elements for repeated exercises and practice during typing training. However, because conventional systems can only detect information keyed in by the learner, those systems evaluate the typing skill of learners using only typing errors and input intervals.

In this study, we propose a method using eyewear to detect the learner’s eye gaze during touch-typing practice to provide effective touch-typing training methods. With this proposed method, learners become conscious of touch typing during typing training and expect to be able to shorten the period required for learning touch typing more than with conventional applications. Our previous work [1] showed the basic experimental result as the first step for the evaluation of learners’ eye gaze direction during typing training by using eyewear. This paper not only provides the necessary background information and our proposed method in detail, but also presents a more detailed analysis of experimental results and the results of an additional experiment to verify the relationship between eye gaze and real touch-typing skills.

The main contributions of this paper are follows:

- We propose a method to detect a learner’s eye gaze during touch-typing skill practice through the use of eyewear, and evaluated the accuracy of our method through analysis of actual measured sensor values.
- Compared with our previous work [1], we improve our eye gaze detection method by conducting feature selection and analyze in detail the accuracy of our method depending on the content of the typing task.
- Finally, we conduct additional experiments to examine the relationship between eye gaze and real touch-typing skills. As an interesting finding, we clarified that typing skills in a real sense cannot be estimated from the gaze, because some users often look at their keyboard unconsciously, even though they have the ability to type without looking at the keys.

## 2. Related Works

### 2.1. Practical Research on Typing Ability

We conducted a broad survey on typing and discovered a significant quantity of practical research on typing ability to investigate. Salthouse [2] experimentally investigated the effect of age on typing ability. His results showed that older typists demonstrated relatively fewer transposition errors than younger typists. Tapping rate and digit-symbol substitution rates for older typists were slower than those for younger typists. Kalava et al. [3] experimentally investigated the typing ability of health care workers along with the spread of electronic medical recordkeeping in recent years. They concluded that most participants did not have adequate typing skills. Feit et al. [4] analyzed characteristics of untrained, self-taught typists from the viewpoint of performance, gaze deployment, and movement strategies. They found that self-taught typists achieve equivalent performance rates with trained typists, even when using fewer fingers. Lubbe et al. [5] found that the more self-regulated learners perform better in keyboarding than less self-regulated learners through a keyboarding test experiment.

### 2.2. Systems of Improvement of Input Speed

Supporting techniques for character input are also studied to improve input speed. Word prediction is frequently used, especially on devices that are burdensome for character input, such as a smartphone. Trnka et al. [6] analyzed the effect of word prediction to improve input speed based on the assumption that accuracy of word prediction strongly affected speed. Anson et al. [7] asserted that word prediction had the potential to reduce typing speed when using a common physical keyboard because the user must look away from the source document to scan the prediction list during typing. Based on this assumption, they investigated the effect of word prediction in a situation when the typist was assigned a task that required looking away from the screen using an input method that requires a user to look at the screen, such as when using a touch-screen on a smartphone. The results indicated that word prediction and completion contributed to the improvement of input speed. Some input technologies for communication were developed for people with severe disabilities. For example, the input method, during which a user selects and inputs characters by using eye movement, dwell time, and blink using eye gaze, is majorly studied [8,9]. These studies especially aimed to realize a fast eye gaze input method by using adjustable dwell time method. Similarly, Sato et al. [10] developed a system that recognizes eye gaze and blinks using image processing.

### 2.3. Study on Typing Training

For improvement of typing skills, learners commonly need to increase training time. Traditionally, companies and individuals actively develop applications to increase motivation for typing practice through the use of game elements and scoring. The gaming application for typing training for visually impaired students is also proposed and evaluated [11]. 

In some studies, typing instruction and evaluation of the instruction is conducted. Weigelt-Marom et al. [12] practically investigated the influence of instruction on touch typing on high school students in the medium to long term. They conducted comparative evaluations between normally achieving students (NA) and students with developmental learning disabilities (LD). The typing speed for NA temporarily decreased at the end of the program, but for LD, it was slightly improved. In addition, when investigating again three months after the end of the program, the results showed that speed improved significantly in both groups when compared with preliminary testing. Donica et al. [13] evaluated the influence of lecture on keyboard inputting for students (general and special education) in grades kindergarten through fifth. 

### 2.4. Supporting Improvement of Typing Ability by Technical Method

In place of the instructor lecture method for typing training, support for improving typing ability through technical methods and interactions has been proposed. An example of such a method is a system that works on muscle memory through use of a device that gives vibration stimulus to the hand. Researchers proposed an environment that passively studies movement typing [14]. 

Yechiam et al. [15] proposed a method in which learners are motivated to watch the screen in order to improve learners’ behavior by discouraging them from looking at the keyboard during typing. In their experiment, participants needed to process secondary tasks that could not be performed without watching the screen during the typing task and were given a penalty when they did not perform the secondary tasks. 

Interactions have been proposed in which a learner’s gaze is detected to determine whether the learner is touch-typing. By applying these methods, an application can visualize the learner’s weaknesses and advocate not looking at a keyboard. Arai et al. [16] proposed a gaze detection method using a face picture of the learner; however, their method could not be used in real-time situations. Imamura et al. [17] also proposed a real-time gaze detection method using a face picture of the learner taken by USB camera. In addition, some studies have been conducted using a web camera to detect eye gaze [18,19,20]. Simple web camera use has become widespread in recent years; standard laptop PCs are commonly equipped with a web camera. Web camera usage is useful as an eye gaze detection method. Zhang et al. [18] proposed an appearance-based accurate eye-gaze detection method in the wild that achieved approximately a 6-degree error for gaze detection, even when evaluated by leave-one-person-out cross-validation. Sewell et al. [19] identified a disadvantage of the camera-based gaze detection method, namely, that detection can be affected by the user’s head movement because the web camera is commonly fixed on the PC or monitor. Furthermore, the camera-based gaze detection method presents invasion of privacy risks.

In contrast with these methods [16,17], our proposed method uses only eyewear, which is a wearable device. Although above-mentioned methods can be implemented easily, they create a concern for privacy because they require photographing learners’ faces. Our method supports the improvement of touch-typing skills using eyewear that is a type of wearable device that has become more common in recent years. Since it is assumed that such devices will become commonplace in the future, touch-typing training can be realized without attaching an eye-tracker or a camera to the PC. Therefore, with our method, there is the possibility for typing training to be carried out anywhere, without concern for the availability of the necessary technology. In addition, because there is no camera to capture facial images, privacy is protected. Our method can track a learner’s high-speed eye movements, while conventional methods using a camera cannot detect the same because of frame rate limitations. In this paper, we discuss the relationship between a learner’s eye gaze and touch-typing skills, which was not verified in previous works.

An important focus of this study is on verifying how to accurately detect the learner’s touch typing using a wearable device, especially one equipped with simple sensors, such as an accelerometer, a gyroscope, and an EOG. Our method is not limited to the JINE MEME; some wearable devices are specialized for gaze detection, such as Pupil Labs [21] (over 300,000 yen) and Tobii Pro Glasses 2 (over 2,000,000 yen). However, such devices have special built-in sensors to measure eye gaze accurately, and they are very expensive for daily use. Further, such devices are not aesthetically suitable for daily wear. In contrast, a simple wearable device like the JINS MEME (over 27,000 yen) is relatively inexpensive and aesthetically appropriate for everyday use. Therefore, it is important to verify how to accurately detect the learner’s touch typing using a simple wearable device.

### 2.5. Touch Typing or Non-Touch Typing

The aim of this study is to develop an interaction to enhance the learner’s touch-typing skills, namely, the ability to type a key without looking at the keyboard. In this section, based on surveys, we consider the benefits of touch typing.

In [4], the difference in gaze movements and typing performance between touch typists and non-touch typists is analyzed. While the 30 participating subjects performed typing tasks, their gaze and behavior was measured using the eye tracker and motion capture to collect data for analysis of the relationship between typing performance and body movements. The data revealed that non-touch typists looked at the keyboard for a longer time than touch typists, but there was no significant difference in typing performance between the two groups. The authors observed that a self-taught strategy that is consistent in its finger-to-key mapping, and minimizes global hand motion, may be easier to acquire and maintain, regarding non-touch typists. At the same time, they note that non-touch typists might not perform as well on interactive tasks that require attention to the display because they need to look at the keyboard.

In [15] the authors describe similar considerations: the performance of touch typists is dependent upon their touch-typing skills. However, skilled touch typists have two advantages. First, touch typists can simultaneously process displays that are far apart (for example, the keys of the keyboard). In contrast, for non-touch typists, the gaze movements involved in searching for a key constitute a serial, time-demanding process. Second, touch typing frees up visual search resources, enabling those resources to be dedicated to reading the source text, which eliminates the need to constantly alternate gaze direction between the screen and the keyboard. However, until typists are skilled in touch typing, visual search is more efficient for input than touch typing; therefore, beginner touch typists tend to select visual searching in daily practice. This fact indicates the difficulty of continuous touch-typing practice.

According to these surveys, although touch typing does not necessarily directly improve input efficiency in daily work, it can be said that it contributes to performance improvement in visual multitask situations, such as when performing subtasks simultaneously with typing. Furthermore, in the process of learning touch typing skills, typists tend to look at the keyboard because visual searching improves input efficiency. Therefore, our proposed interaction to detect touch typing can potentially contribute to the improvement of typing performance in multitasking situations.

## 3. Proposed Method

### 3.1. Eye Gaze Detection by Eyewear

**Figure 1:**
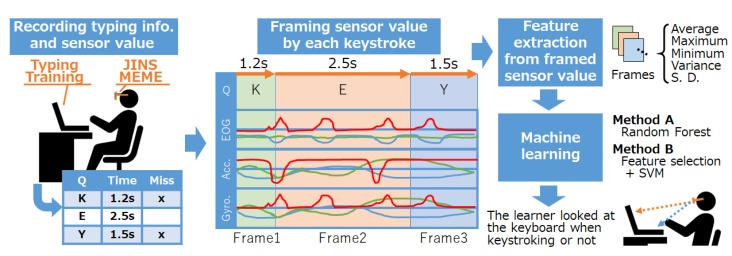
Outline of our proposed system.

Figure 1 shows an outline of our proposed method. Learners wear JINS MEME (https://jins-meme.com/) eyewear while carrying out typing training to detect whether the learner looks at the keyboard for each keystroke by machine learning. The JINS MEME is a wearable device that can measure electrooculography (EOG), acceleration (Acc.), and angular velocity (Gyro.), and has a possibility to measure the learner’s eye gaze and head movements. EOG is measured through the electrodes equipped at the nose pad in a simple way. A gyroscope and acceleration sensor are built in the temple tip position. Sensor data obtained by JINS MEME are composed of a four-axis EOG, three-axis acceleration, and three-axis angular velocity.

Using sensor data for each axis, our method divides that data into frames by each keystroke (as illustrated in Figure 1) and extracts a simple feature vector composed of average values, maximum values, minimum values, variances, and standard deviations. The extracted feature vector selected is used to predict whether the learner was able to touch type. For these purposes, we defined touch typing as whether the learner is looking at the keyboard during typing. Conventional typing training applications can only recognize such user information as typing speed and mistypes. In contrast, our proposed method determines if the learner can touch type when a key is typed once. 

Our proposed method is described as follows:

$$\;\;\;\;\;\;\;C^{n}=f\left(F^{n}\right)$$

where $C^{n}$ is a category whether the learner looked at the keyboard when stroking nth key, and category C is defined as follows:

$$\;\;\;\;\;\;\;C=\{C_{L}|looked\;at\;the\;keyboard,\;\;C_{N}|did\;not\;look\;at\;the\;keyboard\}$$

$F^{n}$ is the feature vector extracted from sensor values $S^{n}$, measured when the (*n* − 1)th and nth key are stroked. The function $f\left(F^{n}\right)$ means trained machine learning model predicts the above category from an input feature vector $F^{n}$. The model $f\left(F^{n}\right)$ is trained using the annotated dataset, which is composed of feature vectors with correct labels, in advance. 

In this study, we adopt two machine learning algorithms: Random Forest (RF) [22] and Support Vector Machine (SVM) [23] to train the model. Random Forest is an ensemble learning method for classification and other tasks that performs by constructing a multitude of decision trees at the training phase and estimating the category for the mode of the classes. One of the characteristics of RF is including feature selection. SVM is a supervised learning algorithm, the main characteristic of which is high generalization performance by the maximization of margin. By applying SVM with a kernel trick, high classification accuracy is achieved. In our method, we deal with high-dimensional features; therefore, we perform feature selection by greedy algorithm for use of SVM. In both methods, feature vectors are normalized in advance. We tried several machine learning algorithms, especially, deep neural networks (DNN), recurrent neural networks (RNN-LSTM, RNN-GRU), and convolutional neural networks (CNN, LeNet, AlexNet, VGG, ResNet). As a result, RF and SVM with feature selection achieved better performance; therefore, we adopted these methods.

The following feedback can also be realized by our proposed method. Conventional typing training applications evaluate typing skills only by analyzing typing speed and errors made. In contrast, our proposed method can realize new dimensions of evaluation about touch typing. We introduce some sample applications of our method. The first idea is to give a penalty. When our method detects that the learner looks at a keyboard during typing, the typing skills score will be decreased as a penalty. As a result, the learner will be conscious of touch typing. The second idea is that a learner cannot go on to the next typing question after looking at the keyboard while typing a key. Therefore, our method can improve a learner’s typing skills by detecting a leaner’s eye gaze during typing.

### 3.2. JINS MEME

JINS MEME [24,25] eyewear used in our proposed method is a device that can measure EOG, acceleration, and angular velocity. Furthermore, the device is comfortable to wear because it feels roughly the same as wearing regular eyeglasses. Based on the concept of “turn your focus on,” JINS MEME is developed to know the state of a wearer’s health, record the levels of exercise, and know the state of mind through eye movement. EOG is measured by simple calculations using three points on the nose pad, and the data is recorded as four-dimensional sensor values. Acceleration and angular velocity are the three axes of x, y, and z, respectively. Therefore, the JINS MEME can measure a total of 10 axes of sensor data. The sampling frequency can be selected; our proposed method for this study uses 50 Hz.

Other research using the JINS MEME has also been conducted. An activity recognition method has been studied conventionally in the research field of ubiquitous computing by using only sensor data obtained with a JINS MEME [26]. Similarly, in [27], a method was developed to estimate the degree of concentration of a user wearing a JINS MEME. In addition, as an application example, Kunze et al. [28] developed a method to recognize the number of words a user read in order to understand reading behaviors. 

The standard API of the JINS MEME implements a method for recognizing blinking; eye moving up, down, left, and right; and a user’s simple activities. Because our defined problem is to recognize whether the learner looks at the keyboard while typing, we adopted a simple technique composed of feature extraction, feature selection, and machine learning using the raw sensor values of EOG, acceleration, and angular velocity of the JINS MEME. Since it is necessary to comprehensively assess motion of the head as well as simple eye gaze movement to detect whether the learner looks at the keyboard, we adopted machine learning. 

## 4. First Experiment: Evaluation of Eye Gaze Detection Accuracy

### 4.1. Details of the Experiments

**Figure 2:**
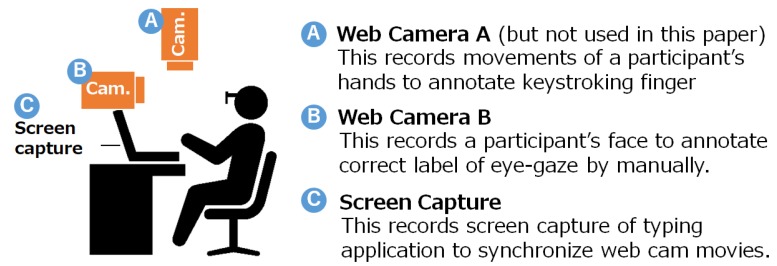
Experimental environment to record annotated data.

**Figure 3:**
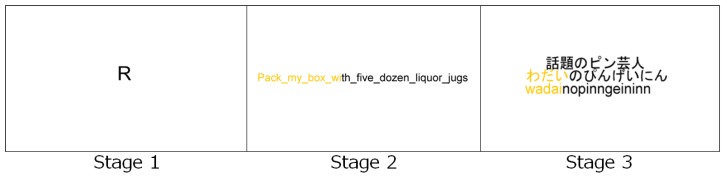
Typing application to perform experiments and to record typing information.

We conducted experiments to evaluate our proposed method. The experimental environment was composed of a common typing training environment and two cameras, as illustrated in Figure 2. In that environment, we asked subjects to wear a JINS MEME and proceed with typing training. Although cameras were not used in our proposed method, they were used to acquire correct data for this evaluation. As shown in Figure 3, we let subjects work on typing tasks on the original application we developed, and recorded the keys pressed and the time required for keystrokes. After the experiment, video recorded by cameras and screen captures from the PC were synchronized, and we manually labeled whether the learner pressed a key without looking at the keyboard.

The experimental procedure consisted of the following steps: (1) give subjects an explanation of research and get consent for handling of personal information; (2) have subjects wear JINS MEME and check for its normal operation; (3) have subjects implement typing tasks. Subjects tried three types of typing tasks as shown in Figure 3. In stage 1, the subjects typed one displayed character taken from a 26-letter alphabet and randomly displayed. They performed three sets of this task. During stage 2, subjects were asked to input 10 English sentences that each constitute a pangram, which is a sentence using every letter of a given alphabet at least once. For example, “The quick brown fox jumps over the lazy dog.” In stage 3, subjects were asked to input 48 Japanese words composed of hiragana characters that were mixed with roughly the same frequency. 

In this experiment, we obtained experimental data from 10 males aged 21 to 23. Every time a key was pressed from the experiment data, a feature vector was calculated, and a correct answer label was manually assigned based on evidence from two video recordings and the synchronized screen capture. We collected a total of 9752 annotated instances from 10 subjects. Although presented typing tasks were the same for each subject, there were some variations among individuals due to typing errors being counted as one keystroke. In cases where two keys were pressed simultaneously, as exception handling, it was assumed that only the correct key had been pressed. 

We evaluated our proposed system using baseline data and results from two evaluation methods. Baseline numbers established the initial rate at which subjects looked at the keyboard during typing, which were used to compare the accuracy difference between baseline and outcome data. One of the evaluation methods is leave-one-subject-out cross-validation (LOSO-CV), which does not include its own data in the training dataset and validates prediction accuracy using each participant’s own data. This evaluation value assigns accuracy assuming an environment in which it is impossible to preliminarily train the dataset using an annotated dataset, which supposes our method cannot calibrate in advance. The other is subject-specific 10-fold CV. Since this evaluation value is evaluated by dividing its own data by ten, it is the estimation of accuracy in the case where the learner’s own correct label is trained in advance, which supposes our method can calibrate in advance, and thus, it is customized for each individual. 

### 4.2. Results of the First Experiment

Table 1 shows the experimental results of detecting whether a subject looked at the monitor or keyboard for each keystroke. 

As a baseline for comparison with our proposed method, for each subject, we show the rate the subject looked at the keyboard in row 1. Rows 2 and 3 (LOSO-CV) are the accuracy estimation compiled by LOSO-CV, and rows 4 and 5 (subject-specific 10-fold CV) give the accuracy estimation compiled by 10-fold CV. These three evaluation indexes are simulated by each keystroke. The label “looked” indicates that the learner looked at the keyboard greater than or equal to once in each case by keystroke. Referring to the results shown in row 1 of the table, learners looked at the keyboard for 52.9% of keystrokes. Although the occurrence of a learner looking at the keyboard varied by individual, the touch-typing skills of the ten subjects were relatively not good in this experiment. Referring to the results shown in rows 2 and 3 of Table 1, using the model that had been trained with other subjects’ dataset, our proposed method could recognize whether the learner looked at the keyboard with an accuracy rate of about 77% for estimation by keystroke. Referring to the results shown in rows 4 and 5 of the table, using the model that had been trained by each learner’s own data, our proposed method could recognize with accuracy rates of about 93% for estimation by keystroke.

Focusing on the accuracy difference between evaluation methods, estimation accuracy was increased from baseline by our method. In cases where our method does not use each learner’s own annotated data for training in the model, the accuracy of estimating looking at the keyboard by keystroke was not sufficient to apply in the real world. However, we confirmed that the estimation accuracy increased by using each learner’s own data to train the model; therefore, it is possible to increase estimation accuracy with our method by using appropriate calibration techniques. This is an interesting result because our method enables the possibility of detecting a learner’s touch typing accurately (accuracy rates of about 93%) in real use by asking the learner to process a calibration at the beginning. 

Focusing on the accuracy difference between learning algorithms, the method adopted by SVM achieved higher accuracy than the one adopted by RF, although the difference was not large. Focusing on the accuracy difference between experimental stages, accuracy in stage 1 was the highest of all stages. Accuracy in stage 1, row 3 was especially high, up to 89.3%. This result indicates that our method can recognize a user’s gaze with accuracy rates of about 90% if the typing questions are composed of a single letter (as shown in stage 1 in Figure 3), even if our method only uses other subjects’ data for training the model. Furthermore, using a learner’s own annotated data for training the model, our method can recognize with an accuracy rate of about 97%.

Table 2 shows the confusion matrices of the results evaluated by LOSO-CV and subject-specific 10-fold CV for each keystroke. “Looked” means that subjects looked at the keyboard, and, “not” means that subjects did not look at the keyboard. Because of deviations in the number of “looked” instances between subjects, the recall of “looked” was high and “not” was relatively low in the result by LOSO-CV. In contrast, all recall and precision are generally high in the result by subject-specific 10-fold CV.

Focusing on estimating touch-typing skills, it is desirable for “looked” to be preferentially detected. On the other hand, missed detections, such as if our method detected “looked” even when the learner did not look at the keyboard, could lower a learner’s motivation. This result also indicates the importance of an appropriate calibration method. Therefore, we will develop the appropriate calibration method; in addition, we will try to investigate the trade-off and improve the estimation accuracy of our method in future work. 

### 4.3. Effect of the Amount of Training Data in Calibration Process

To verify the effects of differing amounts of training data in the calibration process, we additionally compared accuracy rates in the subject-specific case. In this section, we discuss the results, which were determined using our method with RF, for two reasons: (1) the difference in accuracy between RF and SVM is not significant, and (2) feature selection for SVM takes longer to complete.

**Figure 4:**
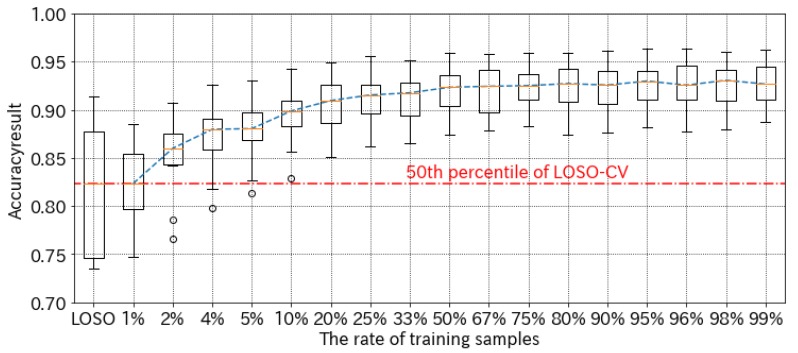
Accuracy comparison between the rate of training samples. These box plots consist of the experimental results of 10 subjects. Because the number of training samples for each subject is about 900, 1% of training samples equals about nine samples.

Figure 4 shows the results of our comparison of training sample accuracy rates. First, we describe the x-axis. As a baseline, LOSO refers to the accuracy rates of 10 subjects evaluated by LOSO-CV, in which the models were trained using other subjects’ data. Other plots (1% to 99%) refer to the rate of training samples picked from the same subject’s data; hence, 99% means the result was evaluated by 100-fold CV, and 1% means that 1% of the samples were used for training and 99% of the samples were used for validation in contrast. As a result, if our method could get 1% of the training samples for test subjects during the calibration process, then our method has achieved roughly the same accuracy as LOSO. Furthermore, 2% of training samples increased accuracy by about 5%, and 10% of training samples increased accuracy by about 7.5%. These results indicate, therefore, that the maximum accuracy for our method is achieved when 50% of the training samples were earned; further, our method did not reduce estimation accuracy to any significant degree, even with limited training data. In our experiments, the average number of key types was 975 (S.D. 74) times, and the average time taken to finish all typing tasks was 665 (S.D. 178) seconds. Therefore, the working time required to obtain calibration data is 26.6 s (88.0% accuracy), 66.5 s (89.9% accuracy), 166.3 s (91.5% accuracy), and 332.5 s (92.3% accuracy). These working times include time to record sensor data, but do not include time to assign correct labels to each recorded data. Thus, learners take additional time to give correct labels for calibration.

### 4.4. Effect of Sensors and Features

**Figure 5:**
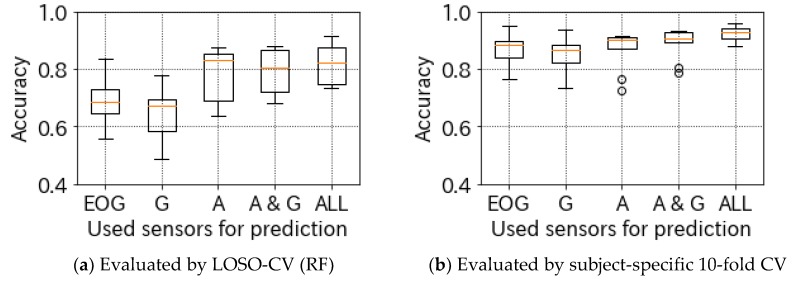
Accuracy distribution using features extracted from each sensor. These box plots consist of the experimental results of 10 subjects.

To verify the effect of features extracted from the EOG sensor, we additionally analyzed performance evaluation. Figure 5 shows the results determined by our method using features extracted from each sensor. The “A” indicates features extracted only from the accelerometer, “G” is gyroscope, “EOG” is electrooculography, “A & G” is the accelerometer and gyroscope, and “ALL” includes features extracted from all sensors. Regardless of the evaluation method (LOSO-CV or user-specific 10-fold CV), ALL features achieved the highest accuracy rates, resulting in the best performance. Although A & G accuracy also indicates relatively better performance, ALL is slightly superior. Comparing the mean value and the standard deviation, A is 74.2 ± 18.8%, A & G is 75.0 ± 18.5%, and ALL is 77.1 ± 18.9%, when evaluated by LOSO-CV; when evaluated by subject-specific 10-fold CV, A is 86.9 ± 6.7%, A & G is 89.1 ± 5.2%, and ALL is 92.5 ± 2.5%. These results show that EOG features slightly affect accuracy, and our method using JINS MEME determined subjects’ touch-typing behavior by head movement and eye gaze comprehensively.

**Figure 6:**
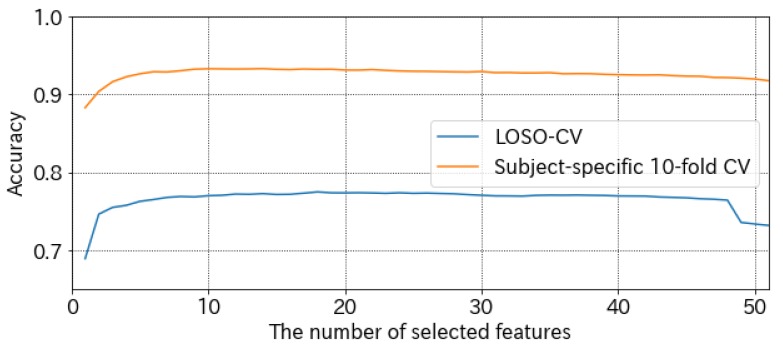
Accuracies change between the number of selected features.

We adopted a feature selection by greedy algorithm and SVM to detect the learner’s touch typing. We discuss the selected features in this paragraph. Figure 6 shows the change in accuracies between the number of selected features. The accuracy of LOSO-CV is best (77.5%) when 18 features are selected. When 14 features are selected, the accuracy of subject-specific 10-fold CV is best (93.3%). According to Figure 6, accuracy rates seem to converge when approximately 10 feature quantities are selected. The accuracy difference between 18-features-selected SVM and all-features-selected SVM is not significant, especially when evaluated by subject-specific 10-fold CV. Therefore, to reduce the burden of the greedy feature selection, removing the feature selection process does not greatly affect accuracy.

Table 3 shows the selected features. Because LOSO-CV used the same features between each subject, we showed the selected order in the table. In contrast, because subject-specific 10-fold CV selected features by each subject, we counted the frequency for each feature, and we showed the 12 most frequently selected features. In subject-specific 10-fold CV, the selected feature is especially different for each subject; further, it tends to look different from LOSO-CV. We considered that this is the cause of the decline in accuracy when our method could not use the subject’s own data in the training phase. 

## 5. Second Experiment: Relationship between a Learner’s Eye Gaze and Touch-Typing Skills

### 5.1. Touch-Typing Difficulty Keys

In this paper, we define whether a learner looked at a keyboard when typing a key as a touch-typing propriety. Our method could detect a learner’s eye gaze with an accuracy of about 93% (when using the learner’s own annotated data). Simply stated, the key at which the learner frequently looks during typing is a touch-typing difficult key. Therefore, by applying our detection method, there is a possibility that a touch-typing difficult key for the learner can be predicted. If touch-typing difficult keys can be detected, it can be presented as one of the skill evaluation indicators of the learner within the typing application, and it can be used as reference information for learner review.

### 5.2. Outline of the Second Experiment

To verify this assumption, after about one month from the first experiment, we performed additional experiments using the same subjects to record touch-typing difficult keys. However, it is not easy to record touch-typing difficult keys; additionally, subjects cannot report on difficult touch-typing difficult keys because they are not able to identify them. We, therefore, performed the additional experiment that involved a subject typing a key in a special environment to record touch-typing difficult keys. We requested the additional experiment to the same subjects; however, one subject could not attend this experiment. Therefore, we collected data from nine subjects.

**Figure 7:**
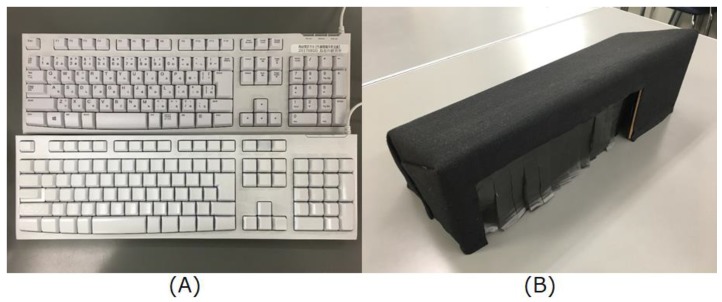
Special environments to collect a subject’s touch-typing difficult keys. (A) is an original blind keyboard (upper one is commercial product and lower one is our blind keyboard) and (B) is covering a keyboard with a black box.

To determine touch-typing difficult keys for each subject, we prepared special environments where a subject must do touch typing, and asked subjects to perform typing tasks. We prepared two special experimental environments where subjects would not be disturbed during typing to the degree possible, namely, that the environment did not hit the subject’s hands, and that the environment did not obstruct screen view. To create one environment, an original blind keyboard was painted white with a spray as shown in Figure 7A. In Figure 7, we placed a common keyboard adjacent to an original blind keyboard to make it clearly understandable. These two keyboards were the same model types, with a slightly different texture as a result of the added paint spray on (A). Protrusions indicating typing home position on the keys F and J were confirmed to be the same on the painted and unpainted keyboard surfaces. The other environment was created by covering a keyboard with a black box as shown in Figure 7B so the subject was unable to look at the keyboard. The subjects placed their hands in the box from the front. In addition, the top plate of the box was made diagonal so as not to block screen view.

We asked nine subjects to perform typing tasks three times for each environment [(A) using an original blind keyboard and (B) covering a keyboard with a black box]. The task involved using the same application as in the previous experimental stage 1 with one letter displayed on the screen to type. We collected data for 26 alphabets, three times, in two environments. We changed the order of use in each environment to prevent the order effect.

Table 4 shows the number of typos for each subject in additional experiments. The column (A) refers to the number of typos when each subject performs tasks using an original blind keyboard, and column (B) refers to the number of typos when each subject performs tasks using a keyboard covered by a black box. Focusing on the average value, the number of typos in (B) is 1.6 times greater than in (A). Focusing on each subject, the difference of typo counts of subjects (D, F, H), where the number of typos in (A) is more than in (B), is not large (4, 4, and 3 times). In contrast, the difference of typo counts of subjects for which the number of typos in (A) is less than in (B), is comparatively large. It is assumed that the cause of the difference is whether the keyboard can be seen. Although subjects could not see key prints, they could see the keyboard in one of the environments (A); therefore, they could remember key arrangement somewhat by seeing the keyboard. On the other hand, the number of typos in (B) was increased by blinding the keyboard. In this paper, the goal of our system is that a user becomes able to type a key without looking not only at the key print, but also at the keyboard; therefore, we adopted the number of typos in (B) as the indicator of touch-typing difficult key.

### 5.3. Discussion

In this section, we discuss touch-typing difficult keys for each learner. Assuming the number of typos in (B) as a correct label of touch-typing difficult keys, we verified whether touch-typing difficult keys could be estimated by using the detection results of our method; however, it could not be achieved. Although we verified that touch-typing difficult keys could be estimated by using features extracted from raw sensor values of JINS MEME, high accuracy could not be achieved.

We formatted the dataset in order to investigate the cause of this result. Since the purpose of this discussion is to investigate the possibility of touch-typing difficult key detection, we count the number of typos for each subject and key. First, we counted the number of typos by each subject and alphabet key when the subject used a keyboard covered by a black box in the additional experiment. This value indicates the frequency of typos when each user cannot look at the keyboard. Second, for each subject and alphabet key, we counted the number of times the subject looked at the keyboard during typing using the first experiment data. Note that the number of times the subject looked at the keyboard is not an estimated value by our method but a correct label we annotated manually. Third, we merged the above two datasets using subject and alphabet key as a key-index to investigate the relationship between the touch-typing difficult key and the number of times the subject looked at the keyboard during typing. Furthermore, to simplify this analysis, we used the label “Touch-typing easy key” when the number of typos was less than two times for each alphabet key, and others as “Touch-typing difficult key.” In addition, we applied the label “Did not look at key” when the number of times the subject looked at the keyboard was less than twice for each alphabet key, and others as “Looked key,” In other words, we accepted failure of up to one.

Table 5 shows the confusion matrix between touch-typing difficult key and the number of times the subject looked at the keyboard. The total number is 234 from 26 alphabet letters for nine subjects. Focusing on the third column (Did not look key), 80.2% of keys (77 of 96) could be typed even if the subject used a keyboard covered by the black box. This result is almost as expected, and it means keys that are correctly typed on a routine basis are the same as touch-typing easy keys. On the other hand, focusing on the second column (Looked key), 45.7% of keys (63 of 138) were typed while looking at the keyboard, even though the subject could type when using the keyboard covered by the black box. As a result, we found that subjects sometimes typed some keys while looking at the keyboard, even though they have the ability to touch type. For these reasons, touch-typing difficult keys could not be estimated by using the detection result of our method. 

As mentioned above, touch-typing difficult keys could not be estimated even if we used the eye gaze information detected through our proposed method. On the other hand, we consider that this result suggests the importance of our proposed system, as it indicates the existence of learners who type keys while looking at the keyboard despite being able to perform touch typing. Such learners merely have a habit of looking at the keyboard, although they actually have the ability to touch type. For such learners, recognizing the look of the keyboard during typing using our proposed method, and assigning a penalty to it, can lead to improvement of habits. Since such learners have the ability to touch type, our proposed system contributes to enhancing learners’ touch-typing skills. 

## 6. Conclusions

In this study, we developed a system to detect a learner’s touch-typing skills by using eyewear in order to support the improvement of those skills. The eyewear JINS MEME measures EOG, acceleration, and angular velocity to detect whether a learner pressed a key by touch typing for each keystroke by machine learning.

In the experiment we conducted with 10 subjects, our proposed method estimated whether a learner could input using touch typing, without looking at the keyboard, with an accuracy of 77.1% when our method did not use the learner’s own labeled data for model training. Further, our method estimated with an accuracy of 92.6% when using the learner’s own labeled data. Owing to the differences between the two, we consider that our method, using appropriate calibration to personalize for the learner, has a possibility to achieve about 90% accuracy without using the learner’s own labeled data. We consider that accuracy can be improved not only by increasing training data, but also by using the subject’s own unlabeled data. Therefore, we plan to investigate application of the domain adaption technique to improve accuracy as a future work.

We conducted additional experiments to analyze whether touch-typing difficult keys can be estimated. As a result of our research, we found touch-typing difficult keys could not be estimated by using eye gaze information. However, this result indicates the existence of learners who type keys while looking at the keyboard despite being able to touch type. Since such learners have the ability to touch type, our proposed system contributes to enhancing learners’ touch-typing skills.

Finally, as a future work, we will analyze how touch-typing skill improvement is influenced by using a typing support application implemented with the proposed method, and we will evaluate the effectiveness of our method by comparison between ours and conventional methods.

## Figures and Tables

**Table 1:** Experimental results of eye gaze (whether a subject looked at a monitor or a keyboard) detection for each keystroke.

		Target Stages			
		All	Stage 1	Stage 2	Stage 3
(1) Baseline		52.9%	45.3%	52.1%	57.6%
(2) LOSO-CV	RF	77.1%	83.5%	76.7%	76.0%
	SVM	77.5%	89.3%	79.3%	79.5%
(3) Subject-specific 10-fold CV	RF	92.6%	95.3%	92.9%	91.7%
	SVM	93.2%	96.4%	94.2%	92.2%

**Table 2:** Confusion matrixes of estimation result by SVM with feature selection.

Confusion Matrix of LOSO-CV					Confusion Matrix of Subject-Specific 10-Fold CV				
		Correct		Precision			Correct		Precision
		Looked	Not				Looked	Not	
Predict	Looked	4516	1552	74.4%	Predict	Looked	4831	332	93.6%
	Not	645	3039	82.5%		Not	330	4259	92.8%
Recall		87.5%	66.2%	77.5%	Recall		93.6%	92.8%	93.2%

**Table 3:** Selected feature name for each evaluation method.

LOSO-CV		Subject-Specific 10-Fold CV	
Feature	Selected Order	Feature	Frequency
EOG_R_std	1	ACC_Y_max	6
ACC_Y_mean	2	Time	5
GYRO_X_std	3	ACC_X_max	5
EOG_H_std	4	EOG_V_mean	4
ACC_Y_min	5	GYRO_X_min	4
ACC_Z_min	6	EOG_V_min	4
GYRO_Y_std	7	EOG_H_mean	4
GYRO_Z_max	8	ACC_Z_max	3
GYRO_X_min	9	EOG_L_std	3
EOG_H_min	10	GYRO_X_max	3
ACC_Y_max	11	GYRO_X_std	3
EOG_R_max	12	EOG_H_std	3

**Table 4:** The number of typos on each special environment while typing tasks.

Subject ID	A	B	C	D	E	F	G	H	I	Avg.
**(A) WHITE**	25	85	45	45	26	76	14	10	27	39.2
**(B) BOX**	35	212	78	41	27	72	50	7	39	62.3
**(B)/(A)**	1.4	2.5	1.7	0.9	1.0	0.9	3.6	0.7	1.4	1.6

**Table 5:** Confusion matrix between touch-typing difficult key and the number of times the subject looked at the keyboard.

	Looked Key	Did Not Look at Key	Sum
**Touch-typing difficult key**	75	19	94
**Touch-typing easy key**	63	77	140
**Sum**	138	96	234
